# Large Language Models Meet Gynecologic Ultrasound: Advancing the Characterization of ADNEXal Masses

**DOI:** 10.3390/jimaging12090462

**Published:** 2026-09-21

**Authors:** Giulia Soccio, Stefania Di Napoli, Paolo Trerotoli, Vera Loizzi, Laura Grazia Zompì, Giuseppe Colonna, Daniele La Forgia, Gennaro Cormio, Francesca Arezzo

**Affiliations:** 1 Department of Interdisciplinary Medicine (DIM), University of Bari “Aldo Moro”, 70124 Bari, Italy; giulia.soccio@uniba.it (G.S.); paolo.trerotoli@uniba.it (P.T.); laura.zompi@uniba.it (L.G.Z.); 2 Gynecologic Oncology Unit, IRCCS Istituto Tumori “Giovanni Paolo II”, 70124 Bari, Italy; vera.loizzi@uniba.it (V.L.); g.colonna@oncologico.bari.it (G.C.); gennaro.cormio@uniba.it (G.C.); f.arezzo@oncologico.bari.it (F.A.); 3 Translational Biomedicine and Neuroscience Department (DiBraIn), University of Bari “Aldo Moro”, Polyclinic of Bari, 70124 Bari, Italy; 4 SSD Radiologia Senologia, IRCCS Istituto Tumori “Giovanni Paolo II”, 70124 Bari, Italy; d.laforgia@oncologico.bari.it; 5 Unit of Obstetrics and Gynecology, Department of Interdisciplinary Medicine (DIM), University of Bari “Aldo Moro”, Polyclinic of Bari, 70124 Bari, Italy

**Keywords:** ultrasound, ovarian cancer, artificial intelligence, large language models, ChatGPT

## Abstract

Ovarian cancer (OC) is the second most common gynecological malignancy and remains one of the leading causes of gynecological cancer-related mortality worldwide. A major clinical challenge is the lack of an accurate and widely applicable strategy for identifying patients at high risk of malignancy at an early stage. In this context, artificial intelligence (AI) has emerged as a promising tool to improve diagnostic performance. Among AI technologies, large language models (LLMs) have recently shown considerable potential in healthcare applications. In this study, we evaluated the diagnostic performance of ChatGPT (GPT-5) in classifying 300 adnexal masses as benign or malignant and compared its performance with that of the IOTA Simple Rules, the ADNEX model, and expert subjective assessment. We also assessed ChatGPT’s ability to predict the most likely histological diagnosis for each lesion. All adnexal masses were described using the International Ovarian Tumor Analysis (IOTA) terminology, and histopathological examination served as the reference standard. Our findings showed that expert subjective assessment achieved the highest overall diagnostic performance for both benign/malignant classification (accuracy 87.3%; 95% CI, 83.0–90.9%) and prediction of the presumed histological diagnosis. ChatGPT A and ChatGPT B reached a sensitivity of 72.3% and 73.5%, a specificity of 74.5% and 75.9%, a positive predictive value of 75.2% and 76.5%, and a negative predictive value of 71.5% and 72.8%, respectively (inconclusive responses counted as misclassifications), with an overall accuracy of 73.3% and 74.7%. After adequate validation, large language models might complement existing decision-support tools for less experienced examiners, without replacing expert evaluation. Their ease of use and reliance on standardized ultrasound descriptors make them accessible to ultrasonographers with varying levels of expertise.

## 1. Introduction

Ovarian cancer (OC) is the fifth most common oncological disease and the fourth leading cause of cancer death in the United States [1], with a 5-year survival rate of approximately 45% [2]. Because early detection significantly reduces mortality, accurately determining whether a pelvic mass is benign or malignant is critical.

Expert sonographers are frequently able to determine the nature of an adnexal mass during the pre-surgical workup with an accuracy of 92%, compared with 82–87% for less experienced examiners [3].

In order to make this assessment objective, reproducible, and universally shared, the definition of a universal language has become necessary. To standardize this assessment universally, the IOTA (International Ovarian Tumor Analysis) group [4] introduced objective ultrasound models, such as the Simple Rules [5] and the ADNEX model (Assessment of Different NEoplasias in the adneXa) [6] (Table 1 and Table 2).

However, the subjective evaluation made by an experienced sonographer has consistently been confirmed as an accurate method to discriminate between benign and malignant lesions [3] with better performance compared with the other ultrasound methods [7].

Since expert sonographers are not always available, AI has emerged as a prominent source of healthcare innovation. Although gynecology relies heavily on imaging, AI implementation in this field is still in its early stages compared to other medical specialties [8].

Multiple studies have demonstrated its validity in the fields of screening [9,10], triage [11,12], diagnosis [13,14], drug development [15,16], treatment [17,18], monitoring [19], and image interpretation [20,21].

In 1999, Timmerman et al. showed that an artificial neural network can be trained to discriminate between a suspected benign or malignant nature of an adnexal mass using some simple ultrasound criteria and some clinical information (e.g., menopausal state and serum level of CA 125) [22].

Acharya et al. developed a machine learning model to automatically discriminate between the diagnosis of benignity and malignancy based on a dataset of 1000 benign and 1000 malignant ultrasound images. This model achieved an accuracy of 99.9%, a sensitivity of 100%, and a specificity of 99.8% [23].

A deep learning model based on a convolutional neural network (CNN) was proposed in the study by Aramendia-Vidaurreta et al. to obtain an automatic classification of adnexal masses, combining the ultrasound characteristics and the age of the patients. This model revealed an overall accuracy of 98.8% with a sensitivity of 98.5% and a specificity of 98.9% [24].

Currently, innovation is driven by large language models (LLMs). These deep learning systems are trained on massive datasets, allowing them to process realistic text and mimic human reasoning and communication. The most widespread variant today is ChatGPT (developed by OpenAI).

Given the exponential growth of AI and the integration of ChatGPT into daily life, evaluating its reliability in healthcare has become an urgent priority.

To date, however, no study has systematically evaluated a state-of-the-art LLM against the validated IOTA instruments (Simple Rules and ADNEX model) and expert subjective assessment on the same consecutive cohort of surgically confirmed adnexal masses described with standardized IOTA terminology. Prior work on artificial intelligence for adnexal masses has focused on image-based approaches, such as neural networks and radiomics [22,23,24], whereas the ability of LLMs to reason over standardized ultrasound descriptors remains essentially unexplored. This is the research gap addressed by the present study.

The aim of our study is to evaluate what could be the prospects of the use of ChatGPT in ultrasound evaluation of adnexal masses, specifically exploring potential and limits in predicting the histological diagnosis. Therefore, this study aims to offer a point of reflection on the new application perspectives of AI such as ChatGPT in the healthcare field and on the possibility that its introduction could facilitate medical work or, conversely, hinder it.

## 2. Materials and Methods

All patients were enrolled at the Gynecology Oncology Unit of the “Giovanni Paolo II” I.R.C.C.S. Cancer Institute in Bari and of the Hospital Polyclinic in Bari between August 2022 and August 2025. All the ovarian masses were analyzed by ultrasound examination according to the IOTA criteria. Clinical and histopathological data were collected from clinical charts.

Ultrasound examinations were performed by one expert operator at each participating center, each having at least 15 years of experience in gynecologic ultrasound.

**Figure 1:**
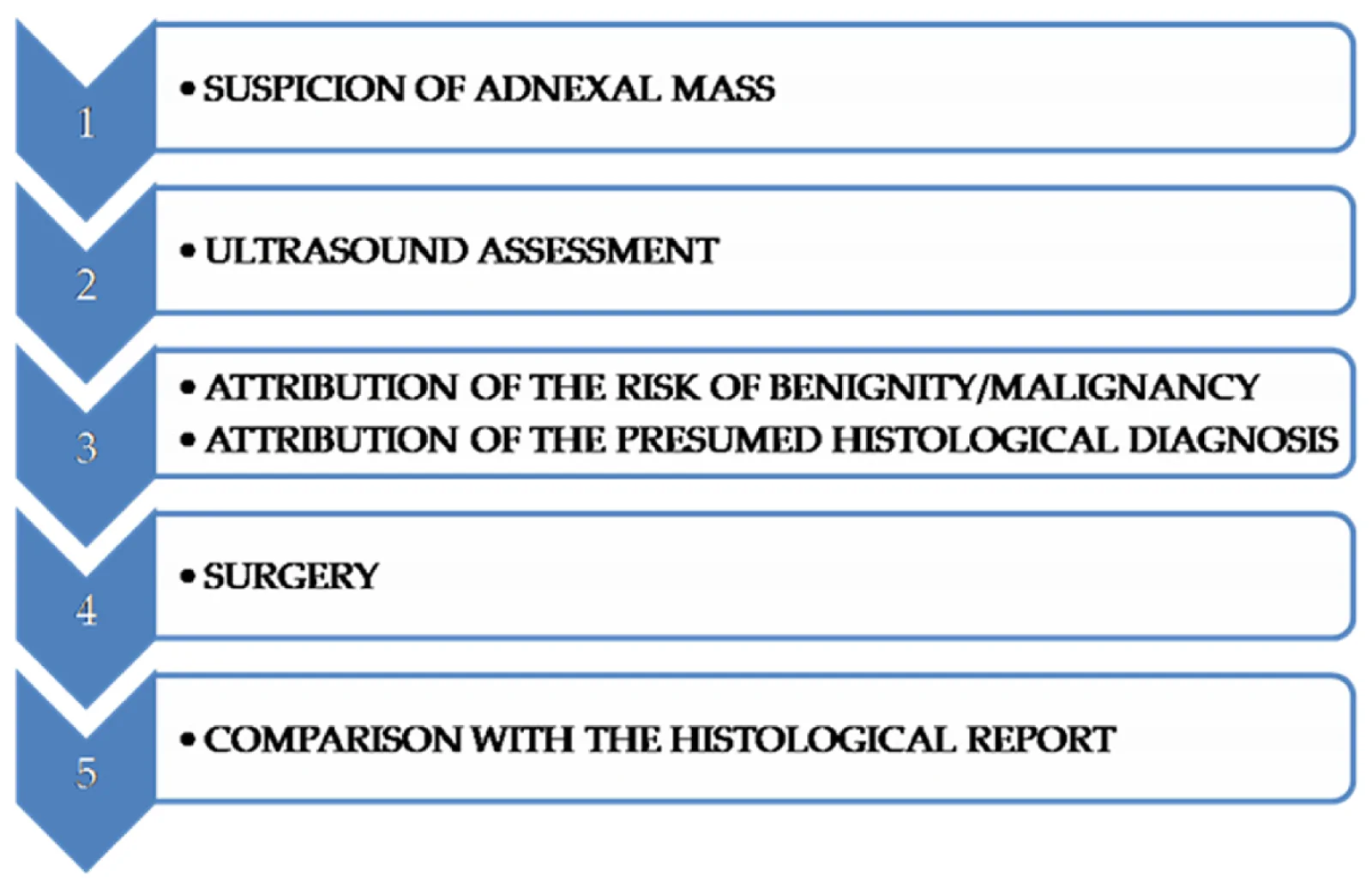
Five-step summary process of the study.

Transabdominal examination was performed with a 3.5–5.0 MHz convex transducer, and transvaginal examination with a 9.0–5.0 MHz broadband transducer (Voluson Expert 22, GE HealthCare, Chicago, IL, USA). The main stages of the study are briefly summarized in Figure 1.

The study was conducted according to the guidelines of the Declaration of Helsinki and the protocol was approved by the Institutional Review Board of “Giovanni Paolo II” I.R.C.C.S. Cancer Institute, Bari, Italy. All patients provided informed consent to use the data for scientific purposes.

**Figure 2:**
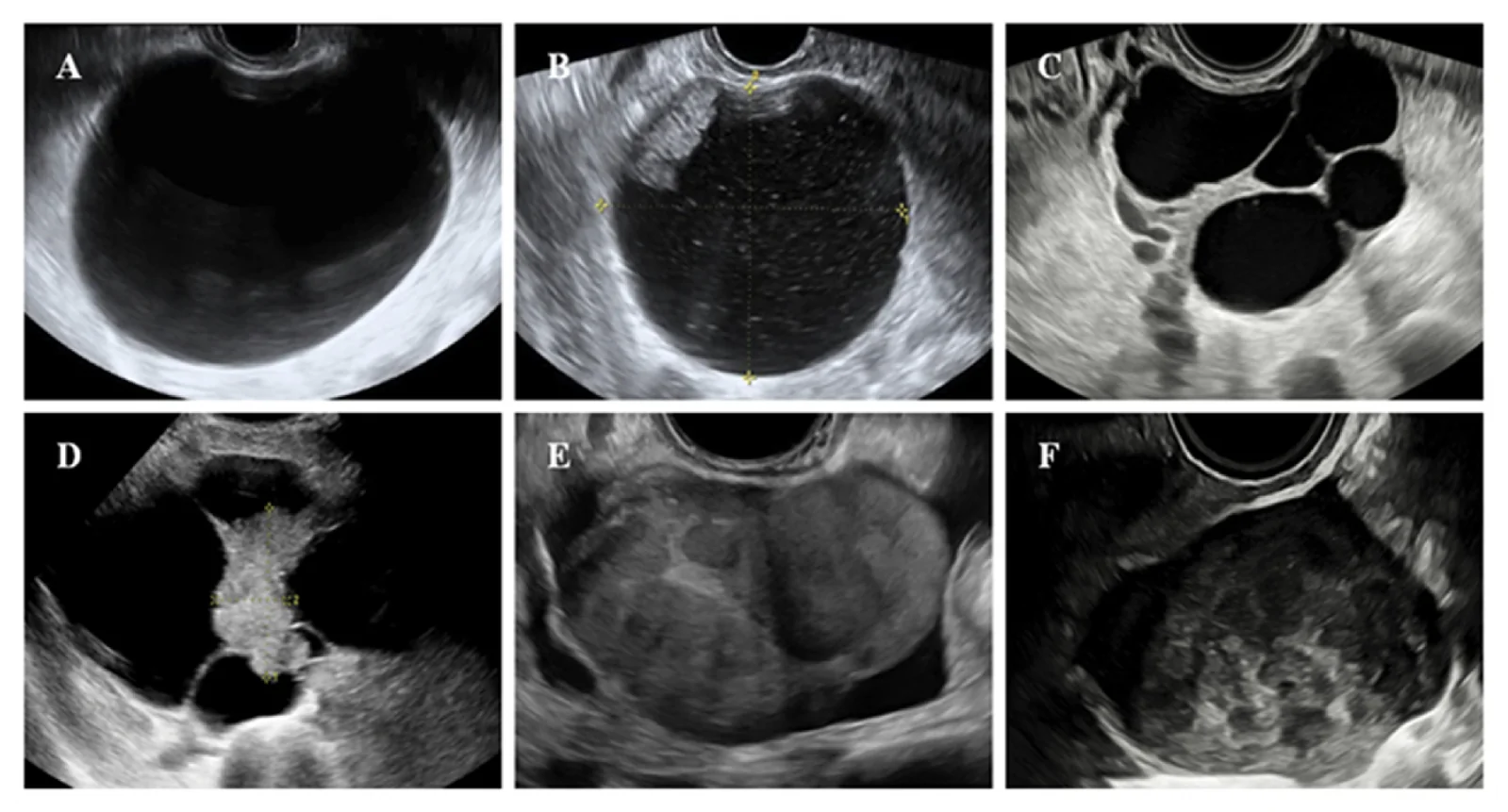
Ultrasound images showing adnexal mass morphologies, according to IOTA terms and definitions: unilocular (**A**), unilocular solid (**B**), multilocular (**C**), multilocular solid (**D**), solid (**E**,**F**).

**Figure 3:**
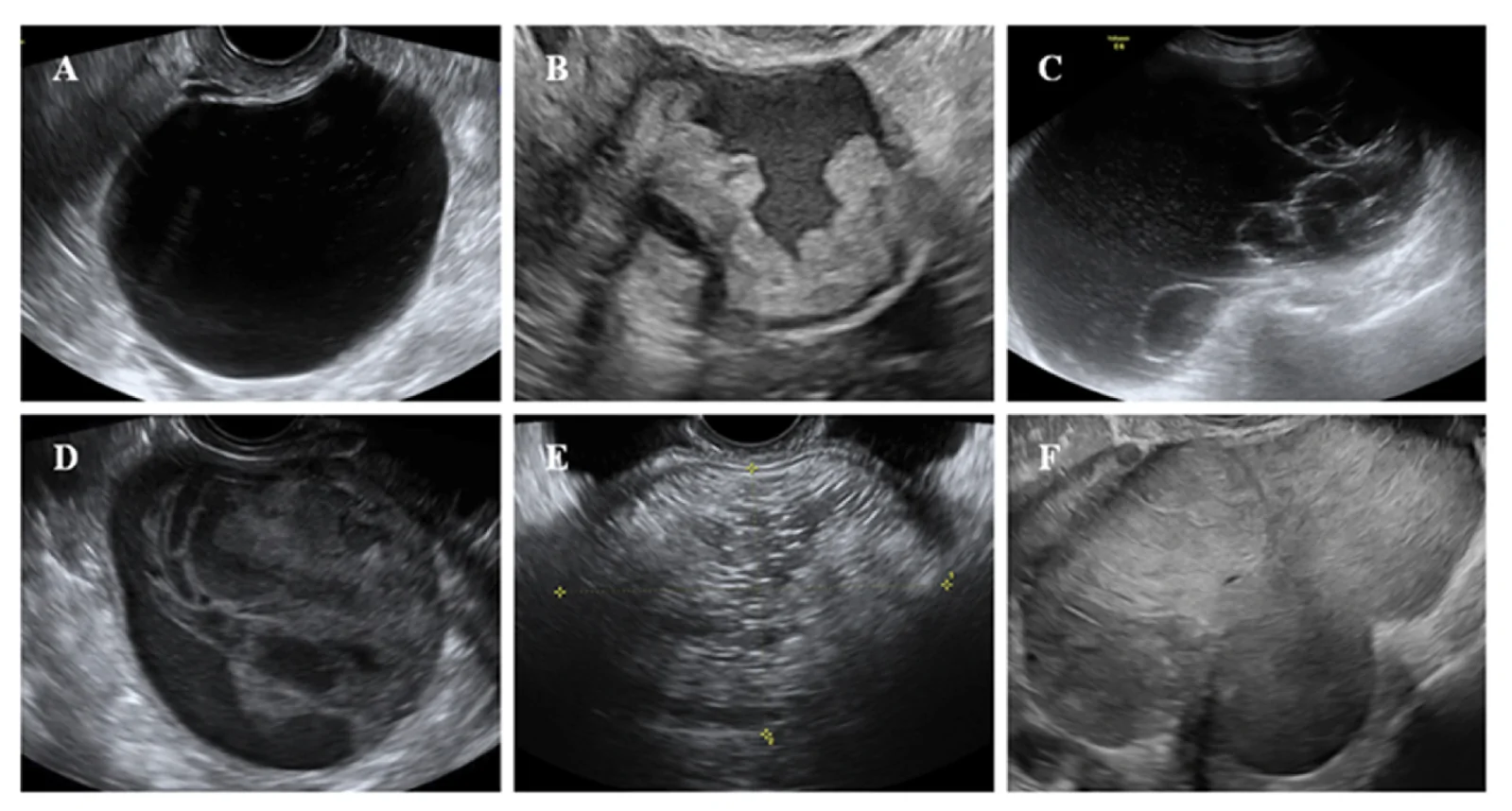
Ultrasound images showing adnexal mass echogenicity, according to IOTA term and definitions: anechoic (**A**), ground glass (**B**), low level (**C**), hemorrhagic (**D**), mixed (**E**), not applicable/solid mass (**F**).

**Figure 4:**
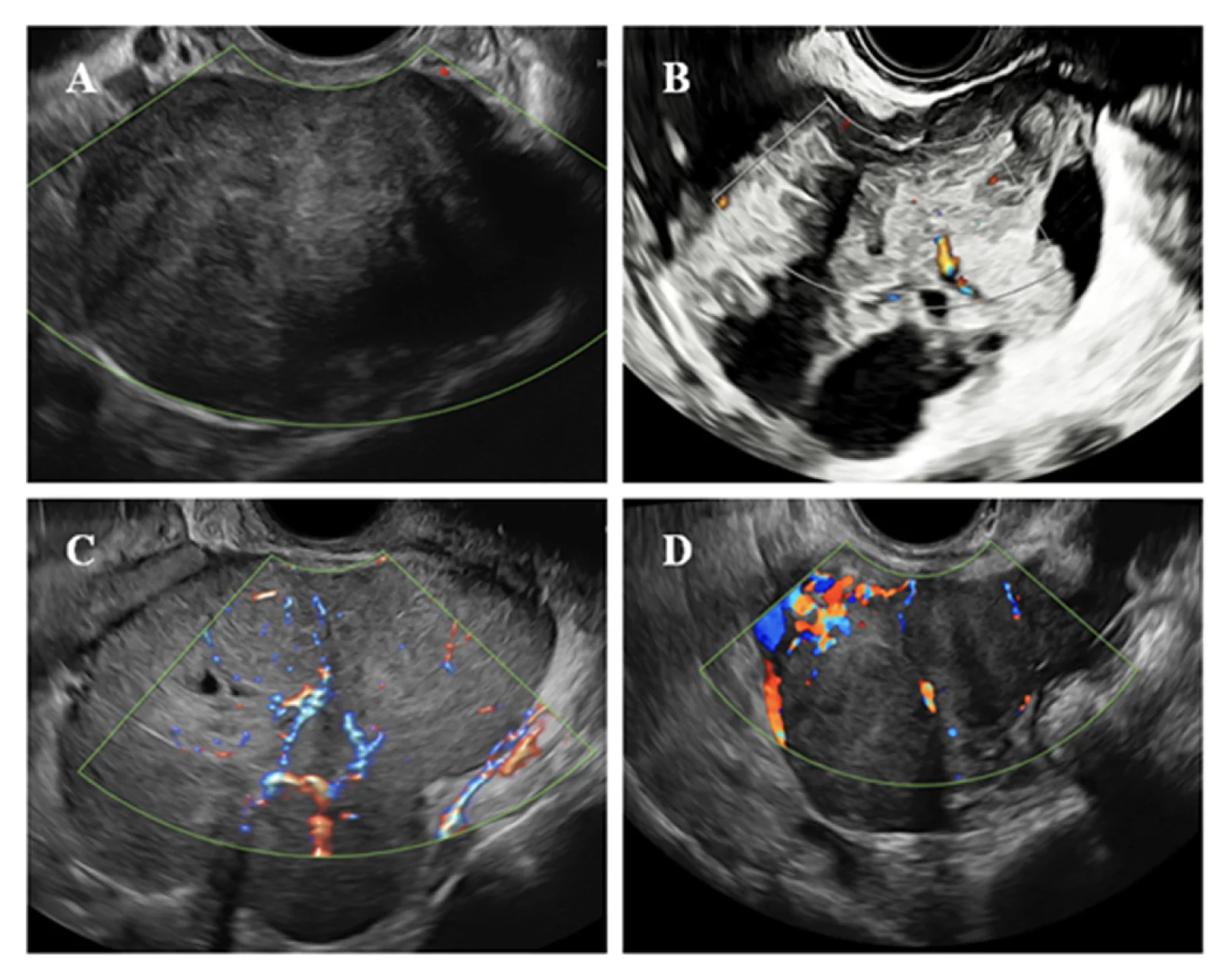
Ultrasound images showing adnexal mass vascularization, according to IOTA terms and definitions: color score 1 when no blood flow is detected (**A**), color score 2 when minimal flow is detected (**B**), color score 3 when moderate flow is detected (**C**), color score 4 when intense flow is detected (**D**).

We collected 339 ultrasound examinations of adnexal masses, which were classified according to their morphology (Figure 2), echogenicity (Figure 3), and vascularization (Figure 4).

In the case of bilateral adnexal masses, the mass with the more complex ultrasound morphology was considered in the analysis. If both masses had similar ultrasound morphology, the larger mass or the one more easily accessible by ultrasound was included.

Inclusion criteria were:

- Suspected adnexal disease for which the patient was referred to centers adherent to the study;
- Execution of a pre-surgical pelvic ultrasound examination;
- Pre-surgical check of serum CA125;
- Patients selected for surgery.

We excluded 39 patients because of missing CA125. All the remaining 300 masses were evaluated by an expert ultrasound examiner who described them using IOTA terminology and assigned the presumptive diagnosis of benignity and malignancy using the Simple Rules, the ADNEX model, and subjective assessment.

The approach to assess the pelvis was, at first, the transvaginal ultrasound, while the transabdominal ultrasound was used just for the masses which cannot be adequately evaluated for dimension, localization, or for patients who were virgo.

Our reference was the histological examination which identified 48.33% benign masses and 51.67% malignant masses.

The OpenAI ChatGPT system (GPT-5) was used to classify adnexal masses as benign or malignant based on standardized ultrasound descriptors.

All cases were submitted through the ChatGPT web interface via a plus subscription, using ChatGPT (GPT-5; model introduced in ChatGPT on 7 August 2025 OpenAI, San Francisco, CA, USA), between 2 September 2025 and 13 October 2025. Web browsing, memory, and all additional tools were disabled. No system instructions other than those reported in Supplementary Material were provided. Sampling parameters (e.g., temperature, top_p) could not be customized in the web interface and were kept at default settings.

Each case was submitted once in a newly opened, independent conversation, so that no information could carry over between cases. The prompt required a predefined response format, and responses were read and coded by G.S. and F.A.; a response was coded as inconclusive when it did not explicitly categorize the lesion as benign or malignant. The complete prompts, including the task instructions, the exact wording and order of the input variables, and the required output format, are reported verbatim in Supplementary Material.

Only fully de-identified, structured ultrasound descriptors and serum CA125 values were entered into the platform; no images, personal identifiers, dates, or free-text clinical notes were transmitted. The information submitted could not, alone or in combination, allow patient re-identification, in compliance with the General Data Protection Regulation (GDPR).

**Figure 5:**
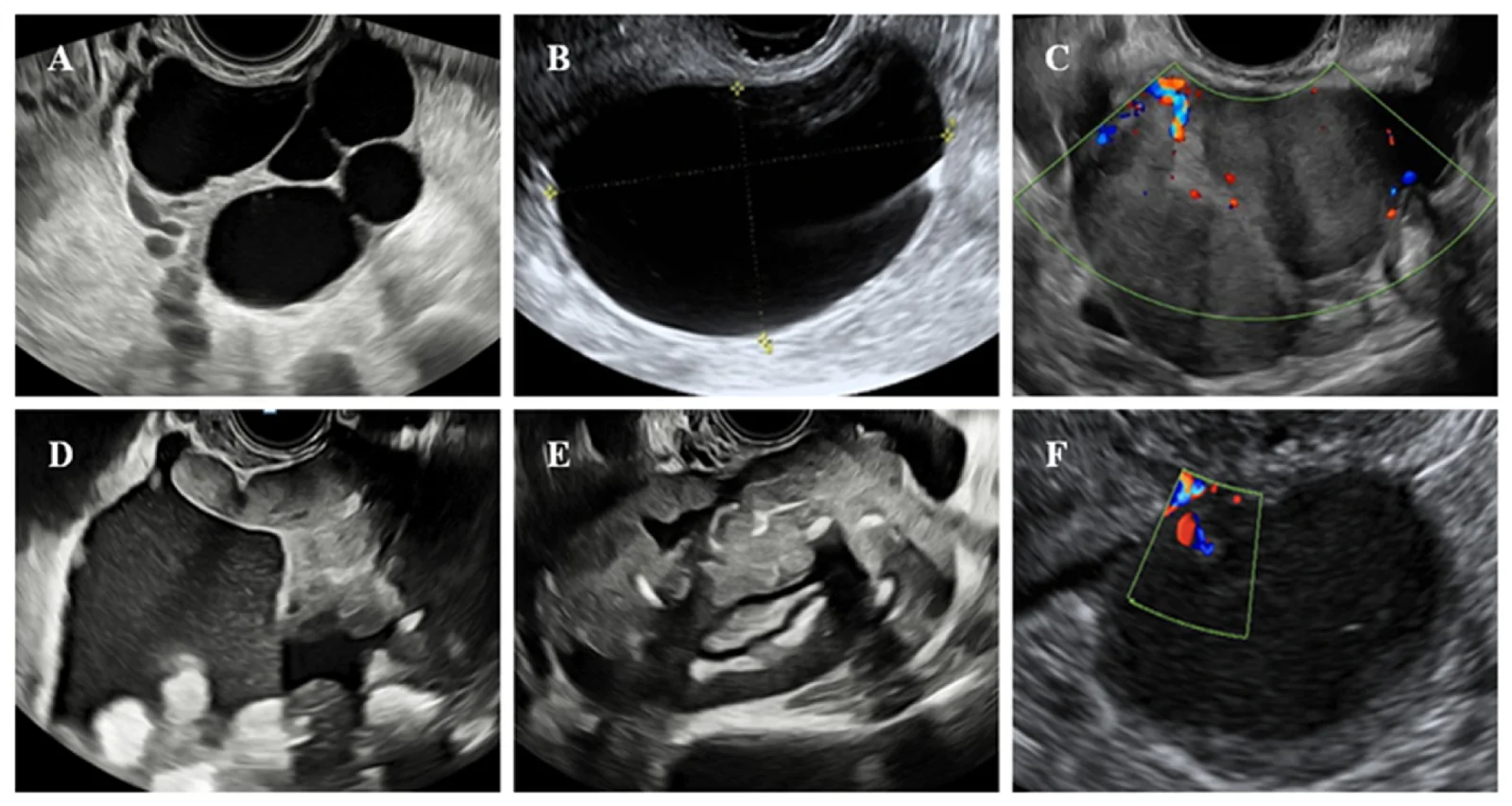
Predefined and additional ultrasound parameters selected to describe the adnexal masses, according to IOTA terminology. (**A**–**C**) show examples of predefined parameters, respectively morphology ((**A**), e.g., multilocular cyst), size (**B**), and vascularization of the lesion (**C**), used in all of the ChatGPT configurations proposed. (**D**) shows the presence of ascites, an additional clinical and ultrasound parameter considered in ChatGPT C configuration. (**E**) represents an example of acoustic shadows, an additional ultrasound parameter considered in ChatGPT D configuration. (**F**) represents a vascularized papilla, an additional ultrasound parameter considered in ChatGPT configuration E.

The main analysis focused on different combinations of parameters (Table 3; Figure 5).

At first, the accuracy of the ChatGPT prompting configurations in the diagnosis of benignity/malignancy was tested against the Simple Rules, the ADNEX model, and subjective assessment. Questions were asked in English describing the adnexal formations according to IOTA terminology and always using the same sequence: morphology, diameter, color score (ChatGPT A) and, then, the additional characterizing element for configurations ChatGPT B, C, D, and E (Table 3). The statement “is a unilocular solid ovarian cyst of 14 mm with a color score 2 and a vascularized papilla more likely benign or malignant?” is an example of the question scheme we followed.

Afterwards, the accuracy of ChatGPT in the determination of the presumed histological diagnosis was tested through standardized questions. The following information was provided: morphology, size, color score, and serum level of presurgical CA125. According to the IOTA Consensus Statement, we chose to add the information of the cystic content for unilocular and unilocular solid morphologies. For multilocular and multilocular solid morphologies, we also added the cystic content and the number of loci. The statement “which is the histological presumed diagnosis for a unilocular solid ovarian cyst with the following features: diameter 199 mm, color score 2, anechoic content, CA125 of 31?” is an example of the question scheme we followed.

The result of the histological examination of the lesion after its surgical removal was used as the reference standard in all phases of the study.

Agreement between the different diagnostic methods was evaluated using Cochran’s Q test for comparing more than two percentages from non-independent samples. If the null hypothesis of equality was rejected, Sheskin’s test for comparing two paired proportions was applied [25].

The accuracy assessment was carried out with particular reference to malignant cases, defining true positives (TP), false negatives (FN), true negatives (TN) and false positives (FP) (Table 4). For each diagnostic method, sensitivity, specificity, positive and negative predictive values, and overall accuracy were determined, along with their respective confidence intervals using the Clopper–Pearson method. Inconclusive ChatGPT responses and cases in which the Simple Rules were not applicable were not treated as test-negative results: in the primary analysis, they were counted as misclassifications, i.e., as false negatives when the lesion was malignant and as false positives when it was benign at histology (intention-to-diagnose approach), and a sensitivity analysis restricted to conclusive classifications was also performed. Differences in sensitivity, specificity, and predictive values between methods were described by comparing point estimates and their 95% confidence intervals.

## 3. Results

The study population consisted of 300 patients, with a median age of 52 years. At the time of the assessment, 162 patients were in menopause, 106 were of fertile age, and for the remaining 32 patients, these data were unavailable. Ascites was present in 41/300 cases.

Table 5 summarizes the characteristics of the examined adnexal formations.

The outcome of the histological examination reported a total of benign formations of 48.33% and a total of malignant formations of 51.67%.

ChatGPT A and ChatGPT B were unable to determine whether the lesion was benign or malignant in 9% (27/300) and 4.33% (13/300) of cases, respectively; to reflect the clinical utility of the configuration, inconclusive responses and cases in which Simple Rules were not applicable were categorized as diagnostic failures (i.e., incorrect outputs), as the configuration failed to recognize the nature of the lesion.

**Figure 6:**
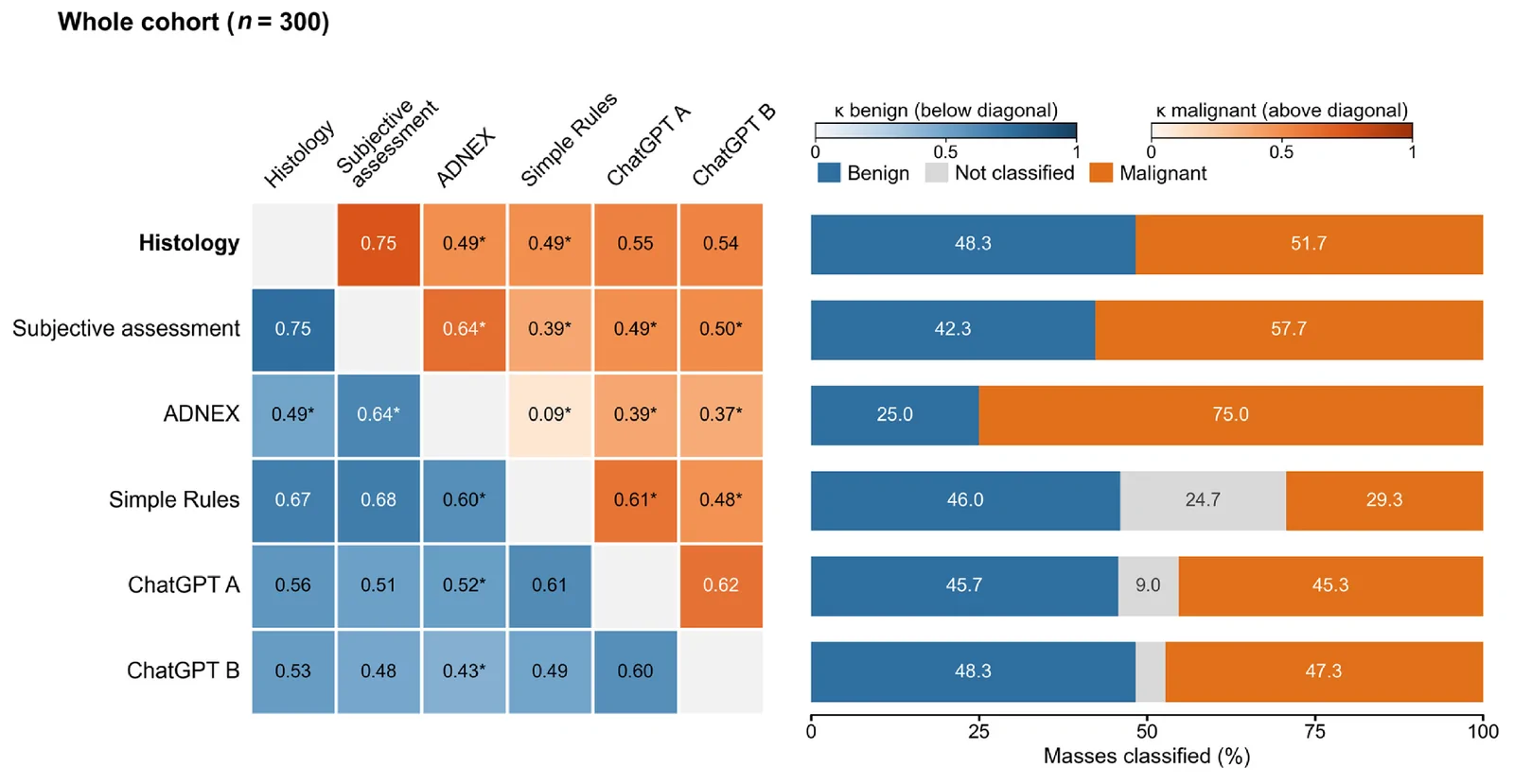
Benign and malignant classifications and pairwise agreement among diagnostic methods applied to the whole cohort (*n* = 300): histology, subjective assessment, ADNEX, Simple Rules and ChatGPT configurations A and B (configurations C and D, evaluated in morphological subgroups, are shown in Figure 7). (**Left**): Pairwise agreement matrix; cells below the diagonal report Cohen’s κ for the benign classification (blue scale) and cells above the diagonal report Cohen’s κ for the malignant classification (orange scale), with darker shading indicating greater agreement. (**Right**): Percentage of masses classified as benign (blue) or malignant (orange) by each method; grey segments correspond to masses without a benign or malignant classification. Asterisks indicate statistically significant pairwise differences in diagnostic proportions following Cochran’s Q test and post hoc comparison of paired proportions (minimum significant difference method). Histopathological examination was used as the reference standard.

The configurations ChatGPT A and B which were assessed for the total study population agree with the histological examination (Figure 6).

The test to compare proportion of diagnosis among different methods showed a statistically significant difference for both benign (Figure 6, Q = 108.04, *p* < 0.001) and malignant (Figure 6, Q = 263.25, *p* < 0.001). The post hoc comparison between pairs was performed through the Sheskin method, which determined the minimum significant difference (MSD) between two paired proportions with a *p*-value lower than 0.05, if the Cochrane test was significant. For malignant, MSD was 10.1%, while for benign, the MSD was 9.5%.

**Figure 7:**
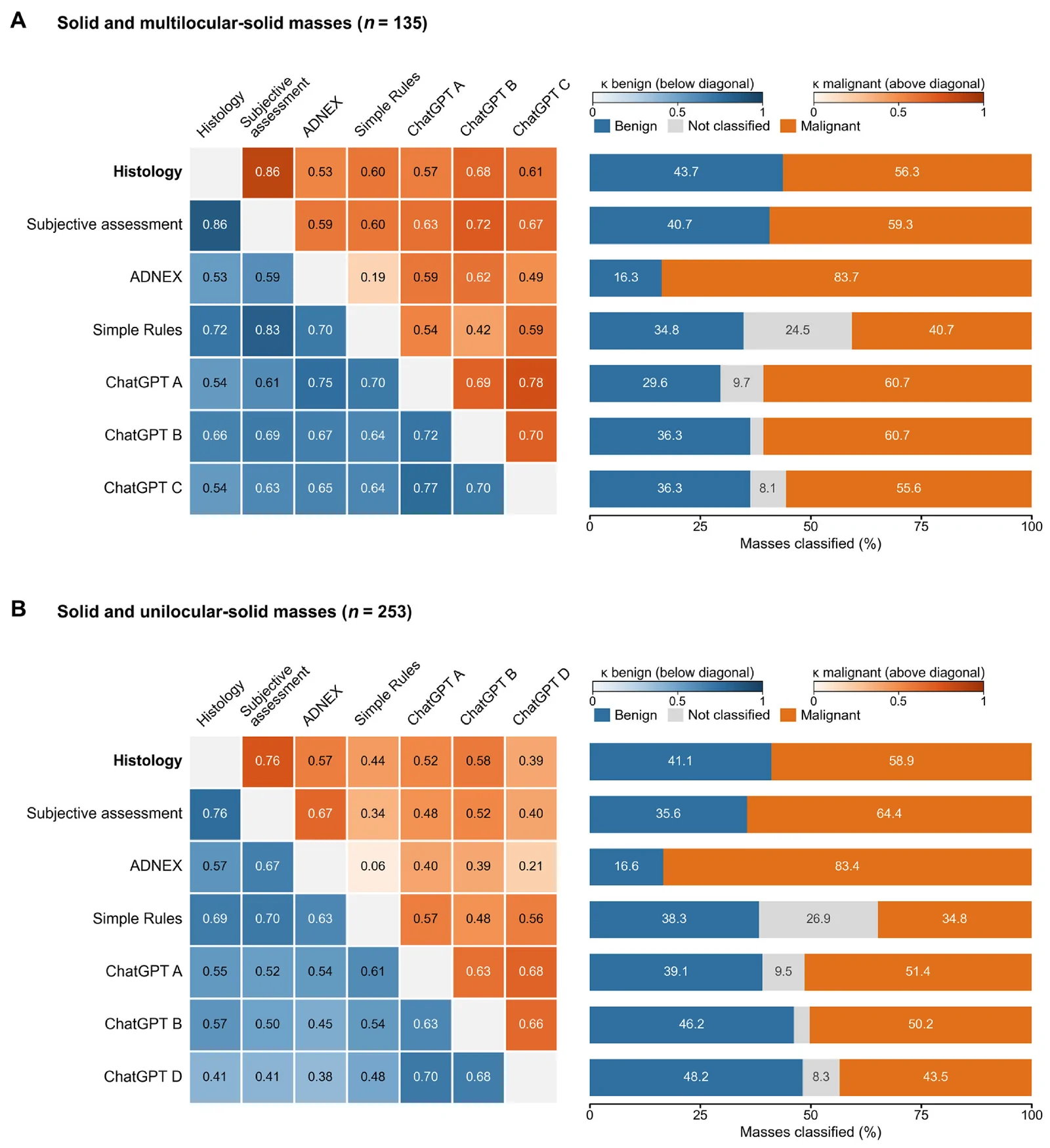
Benign and malignant classifications and pairwise agreement among diagnostic methods within the eligible morphological subgroups. (**A**) Solid and multilocular solid masses (*n* = 135), including ChatGPT configuration C (additional information on ascites). (**B**) Solid and unilocular solid masses (*n* = 253), including ChatGPT configuration D (additional information on acoustic shadows). In each panel, the matrix on the left reports pairwise Cohen’s κ coefficients for the benign classification below the diagonal (blue scale) and for the malignant classification above the diagonal (orange scale), with darker shading indicating greater agreement; the bars on the right show the percentage of masses classified as benign (blue) or malignant (orange) by each method, gray segments corresponding to masses without a benign or malignant classification. Given the different eligible subpopulations, comparisons within subgroups are descriptive. Histopathological examination was used as the reference standard.

In a descriptive evaluation of the subpopulations (prompting configurations ChatGPT C, ChatGPT D, and ChatGPT E), performance was observed when additional parameters were considered beyond the predefined ones (morphology, size, and vascularization). Within its respective subpopulation, ChatGPT C aligned with the histological diagnosis for both malignant and benign lesions. Conversely, ChatGPT D aligned primarily with benign diagnoses, while ChatGPT E yielded a high proportion of inconclusive outputs (Figure 7 for configurations C and D). Since configurations C, D, and E were evaluated in different eligible subpopulations, these comparisons are strictly descriptive and exploratory: a formal assessment of the incremental value of each additional variable would require comparing the baseline and expanded prompts within the same cases.

Comparing the accuracy of the methods examined about the presumed diagnosis benignity vs. malignancy, the subjective assessment results were the most performing overall.

In our study (Table 6), the subjective assessment reported the highest accuracy (87.3%; 95% CI 83.0–90.9%), while the Simple Rules reported the lowest (66.7%; 95% CI 61.0–72.0%).

Analyzing the agreement between the methods, it could be noticed that ADNEX had the highest percentage of malignant diagnosis (225/300 cases, 75%) (Table 7A), while the histological examination reported a percentage of 51.67% cases (155/300 cases). That result could be explained, in our examination, as referable to the nature of the method, which uses a cut-off of 10% to define an adnexal mass as a higher risk of malignancy, with subsequent surgical management. Therefore, in our study, we considered all the masses with an ADNEX score >10% as malignant, but, of course, not all of them were determined to be malignant at the histological diagnosis. This could be the reason why ADNEX overestimated malignant masses in our study population with considerable disagreement compared with other methods. Compared to ADNEX, ChatGPT A and B show a lower number of false positives but higher false negatives, resulting in lower sensitivity but greater specificity.

On the other hand, subjective assessment seems to slightly overestimate the percentage of malignant masses while remaining homogeneous with the histological result. In this regard, the use of an additional method such as ChatGPT to support subjective diagnosis could be of clinical utility.

After analyzing the agreement between methods, we calculated the positive predictive value (PPV), the negative predictive value (NPV), the sensitivity (SE), and the specificity (SP), with their 95% confidence intervals (Table 7B). Since the identification of tumor pathology is of greater clinical use, the focus of this analysis was on the malignant diagnosis.

Subjective assessment showed a higher sensitivity than ChatGPT A (93.5% vs. 72.3%) and ChatGPT B (93.5% vs. 73.5%), with non-overlapping 95% confidence intervals (Table 7B). Configurations ChatGPT A and B showed similar sensitivity (72.3% vs. 73.5%) and specificity (74.5% vs. 75.9%), with largely overlapping confidence intervals.

Regarding predictive values, the main difference between subjective assessment and ADNEX concerned the positive predictive value (83.8% vs. 67.6%, with non-overlapping 95% confidence intervals), which confirms our previous observation related to the ADNEX overestimation of malignancy.

ChatGPT A and B were also similar in terms of positive predictive value (75.2% vs. 76.5%) and negative predictive value (71.5% vs. 72.8%).

The similarity between sensitivity, specificity, and positive and negative predictive values between the two proposed configurations ChatGPT A and B could be due to the similarity between the two methods. In fact, ChatGPT B includes the same ultrasound parameters (morphology, size, and color score) plus the laboratory data (serum level of CA125).

Finally, both predictive values of subjective assessment (PPV 83.8%, NPV 92.1%) were higher than those of ChatGPT A and B, the difference being most evident for the negative predictive value.

The aim of the second phase of the study is to evaluate the feasibility of the use of LLM instead of an expert evaluation in the definition of the presumed histological diagnosis.

The histological reports with the greatest statistical and clinical significance are cystadenoma, cystadenofibroma, teratoma, endometrioma, fibroid, borderline tumor, and invasive carcinoma.

**Figure 8:**
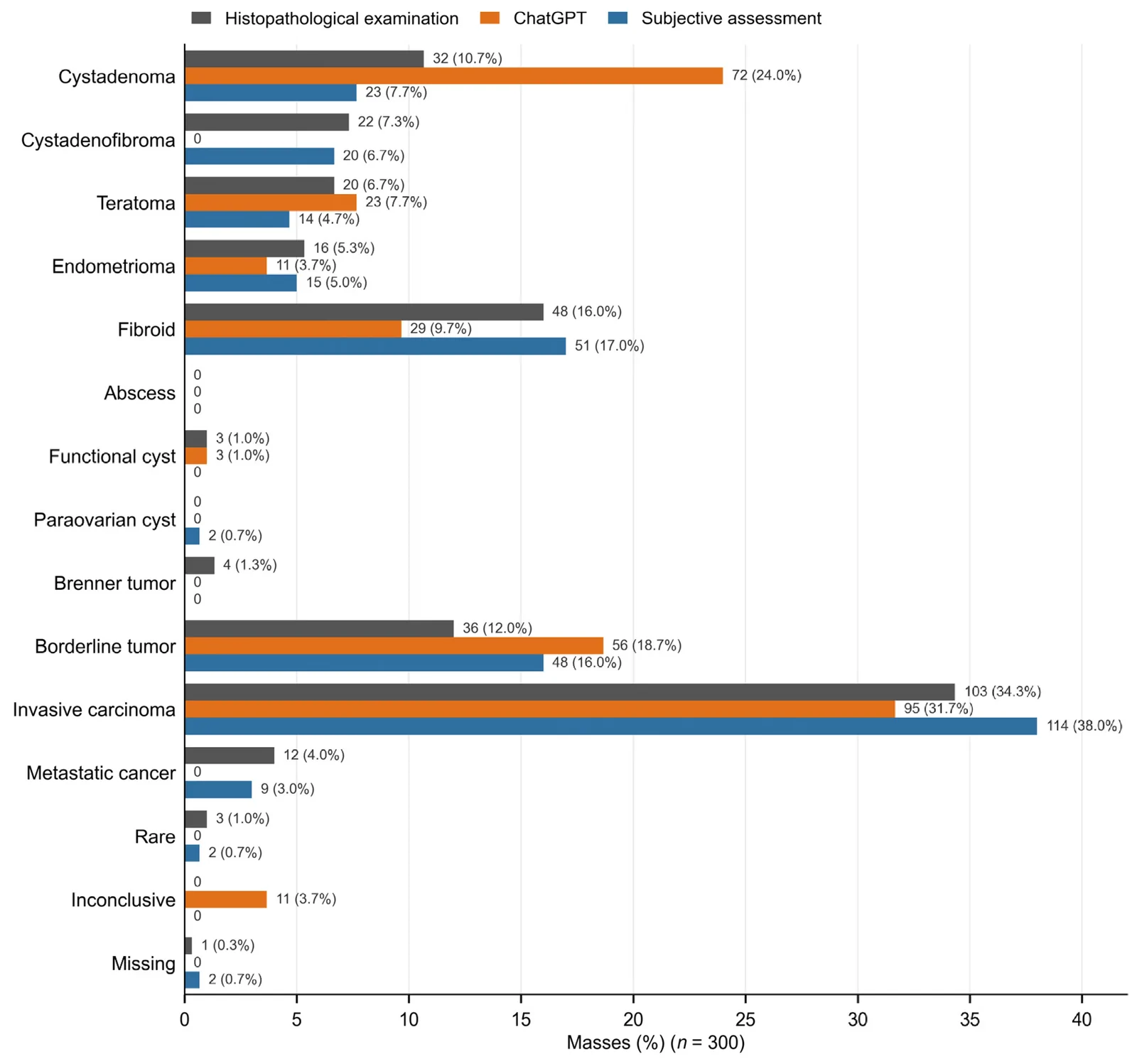
Distribution of the presumed histological diagnoses assigned by ChatGPT and by subjective assessment compared with the histopathological examination (*n* = 300). Bars show the percentage of masses assigned to each diagnostic category; labels report the number of cases with the corresponding percentage (data from Table 8).

Compared with the histological report, the numbers of cases assigned by ChatGPT and by subjective assessment were similar for teratoma and endometrioma, whereas ChatGPT deviated more markedly than subjective assessment from the histological distribution for cystadenoma, cystadenofibroma, fibroid, and borderline tumor (Table 8 and Figure 8).

As can also be seen in Figure 8, ChatGPT seems to overestimate some diagnoses (cystadenoma, borderline tumors, mature teratoma) and underestimate others (cystadenofibroma, invasive carcinomas or metastatic ones, fibroids).

Looking at our results, it is possible to hypothesize that they are due to a limited ability of the system to interpret and contextualize some ultrasound features.

For instance, teratomas and fibroids are both characterized by the ultrasound appearance of acoustic shadows. The discrepancy in the results obtained could be linked to the incorrect interpretation and contextualization of this ultrasound feature, resulting in incorrectly classifying many fibroids as teratomas.

Likewise, cystadenoma and cystadenofibroma could be similar in their ultrasound appearance. Therefore, we could hypothesize that a large proportion of unidentified cystadenofibromas have been misclassified as cystadenomas. Furthermore, misdiagnosed invasive carcinomas may have been classified as borderline cancer. Lastly, ChatGPT does not express an opinion in 3.67% of cases.

## 4. Discussion

OC ranks as the seventh most diagnosed cancer among women worldwide and the second most common gynecological malignancy [26]. Its high lethality is primarily driven by a rapid peritoneal spread and the absence of effective screening programs capable of detecting the disease at an early stage [27]. Identifying accurate tools for early diagnosis and prognosis remains an unmet clinical need [28]. In this scenario, scientific research is increasingly exploring the applications of AI and LLMs, such as ChatGPT, within the gynecological field [29,30,31,32,33,34].

In 2021, the World Health Organization (WHO) published the “Ethics and Governance of Artificial Intelligence for Health” report, establishing a core principle: medical decisions should be made by humans, supported but never replaced by AI. Indeed, the misuse of AI entails significant ethical risks, profoundly impacting:

- Privacy and Cybersecurity: The vulnerability of sensitive patient data.
- Informed Consent and Transparency: The lack of clear protocols regarding how AI is utilized during the diagnostic–therapeutic process.
- Skill Degradation: The risk of a progressive decline in the clinical skills of healthcare professionals due to over-reliance.
- Patient-Guided Misuse: The danger of patients seeking care outside the health system without proper medical surveillance [35].

LLMs have a unique ability to mimic human language. That may result in false, inaccurate, or incomplete statements with a potential negative consequence on patients’ health. Moreover, LLMs could be trained on poor quality or biased data [36].

Despite the growing literature on clinical LLMs, current reviews typically address wide-ranging clinical use cases at the expense of a dedicated focus on disease diagnosis. This has created a critical research gap, as the specific methodologies, strengths, and challenges of deploying LLMs for diagnostic tasks are rarely analyzed in depth.

Existing specialty-specific reviews, focusing on areas such as digestive or infectious diseases [37,38], fail to provide a broader, cross-specialty analysis that incorporates diverse data types, LLM techniques, and clinical diagnostic workflows.

Currently, there are still few studies concerning the application of LLM in the field of gynecology.

A 2024 Italian study revealed that official national guidelines (AIOM) scored significantly higher in clarity, consistency, and completeness compared to answers provided by ChatGPT regarding OC [39].

On the other hand, the study by Cascella et al. highlighted that, despite LLMs’ impressive capabilities, they could obtain poor performance in real settings such as the medical field, where complex high-level thinking is needed [40].

Conversely, ChatGPT demonstrated high concordance with human evaluators when tested in triaging and recommending management interventions for fictitious obstetric and gynecologic emergencies [41].

The study by Ługowski et al. evaluated ChatGPT’s clinical knowledge by assessing its accuracy in answering questions from the Polish Specialty Certificate Examination (SCE) in Obstetrics and Gynecology [42]. They demonstrated that even though ChatGPT is capable of answering very difficult questions, its accuracy is better when solving less complex problems; therefore, the newer version should aid physicians in their daily practice. Moreover, it should be used in English to provide the best accuracy of answers.

To mitigate the risks of misdiagnosis, it is essential to implement systematic processes, ensuring the accuracy of training datasets, and to establish clear procedures for handling situations where the AI’s output conflicts with clinical judgment.

With this study, we aimed to expand the application of LLMs in the field of gynecology, placing a stronger emphasis on diagnostic models. Specifically, we evaluated the diagnostic potential of ChatGPT in the ultrasound assessment of 300 adnexal masses. Although the proposed AI configurations showed a lower overall accuracy than subjective expert assessment, the results are encouraging. ChatGPT offers clear advantages, including easy accessibility and educational utility due to its highly detailed explanations.

However, the model has a critical structural limitation: it cannot interpret ultrasound images directly. It relies entirely on a human operator to provide correct data. In this study, the descriptions were inputted by an expert operator using standardized IOTA terminology. This demonstrates that the accuracy of the AI is strictly dependent on the expertise of the physician, confirming that AI should support, rather than replace, medical judgment.

The lower diagnostic performance observed for ChatGPT is likely explained by its inability to discriminate and appropriately contextualize individual ultrasound features. Interestingly, incorporating additional input parameters, including serum CA-125 levels, vascularized papillary projections, acoustic shadows, and ascites, did not appreciably improve diagnostic accuracy. This finding is not unexpected, as none of these variables is pathognomonic for a specific diagnosis. For example, CA-125 may be elevated in benign conditions such as endometriosis; acoustic shadows may result from fibrous septa, calcifications, or solid tissue, which can be observed in both benign and malignant lesions; vascularized papillary projections are suggestive, but not exclusive, of borderline ovarian tumors; and ascites may occur in non-neoplastic conditions, whereas some patients with ovarian cancer present without ascites. Therefore, these variables require clinical interpretation rather than isolated pattern recognition.

Our text-based approach should also be considered against state-of-the-art artificial intelligence systems that analyze ultrasound images directly. Deep learning models trained on pelvic ultrasound images have achieved expert-level discrimination of ovarian cancer in large multicenter series [43], and a recent systematic review confirmed the rapid growth and overall promising accuracy of image-based artificial intelligence in gynecologic oncology [44]. Radiomics-based machine learning has likewise shown good diagnostic performance for adnexal masses [45]. Compared with these systems, an LLM working on standardized IOTA descriptors currently offers lower discriminative performance, but it requires no imaging pipeline, is immediately available in clinical settings, and leverages the same standardized lexicon already used in routine reporting. The two approaches are therefore complementary, and multimodal systems combining direct image analysis with structured descriptors represent a natural next step.

The application of LLMs in the clinical management of adnexal masses and ovarian cancer is supported by the recent literature across both pre-diagnostic screening and ultrasound stratification. LLM-based natural language processing has proven effective in extracting non-coded symptom signatures from unstructured electronic health records to support early cancer detection [46]. Additionally, LLMs have demonstrated high accuracy and reliability in standardizing O-RADS categorization directly from free-text ultrasound reports, highlighting their utility as clinical decision-support tools [47].

The principal limitation associated with the lower diagnostic accuracy of ChatGPT is the potential for inappropriate clinical management of adnexal masses. False positive results may lead to unnecessary surgery, whereas false negative classifications may delay referral and treatment of ovarian malignancies. Therefore, the probability of benignity or malignancy generated by ChatGPT should always be interpreted together with the patient’s clinical history, physical examination, laboratory findings, and comprehensive ultrasound assessment.

Future studies should evaluate ChatGPT in real-world clinical settings, where more comprehensive clinical information is available than the limited set of ultrasound descriptors and additional variables included in the present study. Moreover, multimodal artificial intelligence models combining direct ultrasound image analysis with structured clinical data should be explored. Multicenter validation studies are also warranted to assess the generalizability of ChatGPT across different imaging platforms and healthcare settings. Finally, domain-specific fine-tuning of large language models using curated gynecological ultrasound reports may further improve diagnostic performance.

Although the current evidence is encouraging, the integration of LLMs into routine clinical practice will require further improvements in model performance through training on larger, high-quality datasets, together with the development of robust ethical and regulatory frameworks addressing transparency, privacy, data security, and informed consent. Most importantly, LLMs should be regarded as decision-support tools that complement, rather than replace, clinical expertise. Importantly, we acknowledge that evaluating each case with a single prompt is a limitation of the present study as it does not capture output variability; accordingly, ongoing and future trials will incorporate repeated queries to formally assess response stability.

## 5. Conclusions

Expert subjective assessment remains the most accurate approach for the ultrasound characterization of adnexal masses. Although ChatGPT demonstrated lower diagnostic performance, it showed potential as an additional decision-support aid for less experienced examiners, to be used alongside validated IOTA instruments and never as a substitute for referral to an expert sonographer. Its performance depends on the use of standardized IOTA terminology and should always be interpreted in conjunction with clinical evaluation rather than as a stand-alone diagnostic method. Future research should focus on the paired evaluation of prompting strategies on identical case sets, the formal assessment of response stability across repeated queries, prospective multicenter validation, the comparison of different LLMs, including open-weight models deployable on premise for privacy reasons, and studies measuring the impact on clinician workload and diagnostic efficiency rather than diagnostic agreement alone.

## Figures and Tables

**Table 1:** IOTA Simple Rules.

M-RULES		B-RULES	
M1	Irregular solid tumor	B1	Unilocular
M2	Presence of ascites	B2	Presence of a solid component with a maximum diameter < 7 mm
M3	At least 4 papillary structures	B3	Presence of “acoustic shadows”
M4	Irregular multilocular solid tumor with a maximum diameter ≥ 100 mm	B4	Regular multilocular tumor with a maximum diameter < 100 mm
M5	Rich vascularization (color score 4)	B5	Absence of vascularization (color score 1)

**Table 2:** IOTA ADNEX model (Assessment of Different NEoplasias in the adneXa).

Clinical Variables	Patient’s Age
Ultrasound variables	Ultrasound performed at a cancer center (yes/no)
	CA125 measurement (optional)
	Maximum lesion diameter (in millimeters)
	Maximum solid part diameter (in millimeters)
	More than ten locules (yes/no)
	Number of papillae (0, 1, 2, 3, >3)
	Presence of acoustic shadows (yes/no)
	Presence of ascites (yes/no)

**Table 3:** Summary of the ChatGPT prompting configurations (A–E) used to evaluate the benignity or malignancy of an adnexal mass based on combinations of ultrasound, laboratory, and clinical data.

Configuration	Adnexal Masses	Number of Patients	Predefined US Parameters			Additional Parameter
A	All	300	Morphology	Size	Color score	None
B	All	300	Morphology	Size	Color score	Serum CA125
C	Solid, multilocular solid	135	Morphology	Size	Color score	Ascites
D	Solid, unilocular solid	253	Morphology	Size	Color score	Acoustic shadows
E	Unilocular solid, multilocular solid	134	Morphology	Size	Color score	Vascularized papilla

**Table 4:** Accuracy assessment: definition of true positive, false positive, false negative, and true negative used in the analysis. Histological examination is the reference standard.

	Malignant at Histology	Benign at Histology
Classified as malignant by the method	True positive (TP)	False positive (FP)
Classified as benign by the method	False negative (FN)	True negative (TN)

**Table 5:** Main ultrasound features of the adnexal masses examined (*n* = 300). Values are numbers of cases, with percentages of the total population in parentheses.

Feature	Category	*n* (%)	Malignant (tot = 155)	Benign (tot = 145)
Side	Unilateral	233 (77.7)	107 (69.0)	126 (86.9)
	Bilateral	66 (22.0)	47 (30.3)	19 (13.1)
	Uncertain	1 (0.3)	1 (0.7)	0 (0)
Origin	Ovary	276 (92.0)	145 (93.6)	131 (90.3)
	Salpinx	6 (2.0)	4 (2.6)	2 (1.4)
	Paraovarian	9 (3.0)	1 (0.6)	8 (5.5)
	Paratubal	0 (0)	0 (0)	0 (0)
	Uncertain	9 (3.0)	5 (3.2)	4 (2.8)
Morphology	Unilocular	23 (7.7)	0 (0)	23 (15.9)
	Unilocular solid	126 (42.0)	76 (49.1)	50 (34.5)
	Multilocular	16 (5.3)	3 (1.9)	13 (9.0)
	Multilocular solid	8 (2.7)	3 (1.9)	5 (3.4)
	Solid	127 (42.3)	73 (47.1)	54 (37.2)
Echogenicity	Anechoic	65 (21.7)	26 (16.8)	39 (26.9)
	Ground glass	32 (10.7)	16 (10.3)	16 (11.0)
	Low level	57 (19.0)	34 (21.9)	23 (15.9)
	Hemorrhagic	0 (0)	0 (0)	0 (0)
	Mixed	14 (4.7)	1 (0.7)	13 (9.0)
	Not applicable (solid)	129 (43.0)	75 (48.4)	54 (37.2)
	Uncertain	3 (1.0)	3 (1.9)	0 (0)
Papillae	Present	63 (21.0)	33 (21.3)	30 (20.7)
	Absent	237 (79.0)	122 (78.7)	115 (79.3)
Vascularized papillae	Yes	37 (12.3)	26 (16.8)	11 (7.6)
	No	26 (8.7)	7 (4.5)	19 (13.1)
	No papillae	237 (79.0)	122 (78.7)	115 (79.3)
Acoustic shadows	Present	93 (31.0)	23 (14.8)	70 (48.3)
	Absent	207 (69.0)	132 (85.2)	75 (51.7)
Color score	No flow (1)	117 (39.0)	19 (12.3)	98 (67.6)
	Minimal flow (2)	56 (18.7)	24 (15.5)	32 (22.1)
	Moderate flow (3)	54 (18.0)	41 (26.4)	13 (8.9)
	Very strong flow (4)	73 (24.3)	71 (45.8)	2 (1.4)
Ascites	Present	41 (13.7)	39 (25.2)	2 (1.4)
	Absent	259 (86.3)	116 (74.8)	143 (98.6)

**Table 6:** Accuracy of the methods examined (subjective assessment, Simple Rules, ADNEX, ChatGPT A, and ChatGPT B) concerning the presumed diagnosis benignity vs. malignancy referring to the total population. The subjective assessment results were the best performing overall.

Method	Correctly Classified Benign (*n* = 145)	Correctly Classified Malignant (*n* = 155)	Total Correct (*n* = 300)	Accuracy, % (95% CI)
Subjective assessment	117	145	262	87.3 (83.0–90.9)
ADNEX	72	152	224	74.7 (69.3–79.5)
Simple Rules	117	83	200	66.7 (61.0–72.0)
ChatGPT A	108	112	220	73.3 (67.9–78.3)
ChatGPT B	110	114	224	74.7 (69.3–79.5)

Note: Only explicit benign or malignant classifications were counted as correct; inconclusive responses and cases in which the Simple Rules were not applicable were counted as incorrect.

**Table 7:** Diagnostic performance of subjective assessment, Simple Rules, ADNEX, ChatGPT A, and ChatGPT B in the total population of 300 patients, with histopathology as the reference standard. (**A**) reports true positives, false positives, false negatives, and true negatives; inconclusive ChatGPT responses and cases in which the Simple Rules were not applicable are reported separately and were not counted as test-negative. (**B**) reports sensitivity, specificity, positive predictive value (PPV), and negative predictive value (NPV) as % (*n*/*N*) with 95% confidence intervals.

(**A**)					
**Method**	**True Positive**	**False Positive**	**False Negative**	**True Negative**	**Inconclusive or Not Applicable, *n* (Malignant; Benign)**
Subjective assessment	145	28	10	117	0
Simple Rules	83	28 (5 + 23)	72 (21 + 51)	117	74 (51; 23)
ADNEX	152	73	3	72	0
ChatGPT A	112	37 (24 + 13)	43 (29 + 14)	108	27 (14; 13)
ChatGPT B	114	35 (28 + 7)	41 (35 + 6)	110	13 (6; 7)
(**B**)					
Method		Sensitivity, % (*n*/*N*) (95% CI)	Specificity, % (*n*/*N*) (95% CI)	PPV, % (*n*/*N*) (95% CI)	NPV, % (*n*/*N*) (95% CI)
Subjective assessment		93.5 (145/155) (88.5–96.9)	80.7 (117/145) (73.3–86.8)	83.8 (145/173) (77.5–89.0)	92.1 (117/127) (86.0–96.2)
Simple Rules		53.5 (83/155) (45.4–61.6)	80.7 (117/145) (73.3–86.8)	74.8 (83/111) (65.6–82.5)	61.9 (117/189) (54.6–68.9)
ADNEX		98.1 (152/155) (94.4–99.6)	49.7 (72/145) (41.3–58.1)	67.6 (152/225) (61.0–73.6)	96.0 (72/75) (88.8–99.2)
ChatGPT A		72.3 (112/155) (64.5–79.1)	74.5 (108/145) (66.6–81.4)	75.2 (112/149) (67.4–81.9)	71.5 (108/151) (63.6–78.6)
ChatGPT B		73.5 (114/155) (65.9–80.3)	75.9 (110/145) (68.1–82.6)	76.5 (114/149) (68.9–83.1)	72.8 (110/151) (65.0–79.8)
Sensitivity analysis restricted to conclusive classifications (inconclusive or not applicable cases excluded)					
Simple Rules (*n* = 226)		79.8 (83/104) (70.8–87.0)	95.9 (117/122) (90.7–98.7)	94.3 (83/88) (87.2–98.1)	84.8 (117/138) (77.7–90.3)
ChatGPT A (*n* = 273)		79.4 (112/141) (71.8–85.8)	81.8 (108/132) (74.2–88.0)	82.4 (112/136) (74.9–88.4)	78.8 (108/137) (71.0–85.3)
ChatGPT B (*n* = 287)		76.5 (114/149) (68.9–83.1)	79.7 (110/138) (72.0–86.1)	80.3 (114/142) (72.8–86.5)	75.9 (110/145) (68.1–82.6)

Note: Primary analysis according to an intention-to-diagnose approach. Inconclusive ChatGPT responses and cases in which the Simple Rules were not applicable were not counted as test-negative; they were considered misclassifications, i.e., false negatives when the lesion was malignant and false positives when it was benign at histology (in parentheses: conclusive misclassifications + inconclusive or not applicable cases). This is the same counting rule used in Table 6, so that (TP + TN)/300 corresponds to the accuracy reported there. The 95% confidence intervals were calculated with the Clopper–Pearson method. Upper rows: Intention-to-diagnose analysis (*N* = 155 malignant and 145 benign masses for sensitivity and specificity), in which inconclusive or not applicable results were counted as misclassifications. Lower rows: Sensitivity analysis excluding inconclusive ChatGPT responses and cases in which the Simple Rules were not applicable; subjective assessment and ADNEX always provided a conclusive classification and are therefore reported only once. PPV, positive predictive value; NPV, negative predictive value.

**Table 8:** Comparison between methods (subjective assessment vs. ChatGPT) in the detection of the presumed histological diagnosis. The histological report is the reference standard. Values are numbers of cases, with percentages of the total population in parentheses.

Histopathological Diagnosis	Histological Report, *n* (%)	ChatGPT, *n* (%)	Subjective Assessment, *n* (%)
Cystadenoma	32 (10.67)	72 (24)	23 (7.67)
Cystadenofibroma	22 (7.33)	0 (0)	20 (6.67)
Teratoma	20 (6.67)	23 (7.67)	14 (4.67)
Endometrioma	16 (5.33)	11 (3.67)	15 (5)
Fibroid	48 (16)	29 (9.67)	51 (17)
Abscess	0 (0)	0 (0)	0 (0)
Functional cyst	3 (1)	3 (1)	0 (0)
Paraovarian cyst	0 (0)	0 (0)	2 (0.67)
Brenner tumor	4 (1.33)	0 (0)	0 (0)
Borderline tumor	36 (12)	56 (18.67)	48 (16)
Invasive carcinoma	103 (34.33)	95 (31.67)	114 (38)
Metastatic cancer	12 (4)	0 (0)	9 (3)
Rare	3 (1)	0 (0)	2 (0.67)
Inconclusive	0 (0)	11 (3.67)	0 (0)
Missing	1 (0.33)	0 (0)	2 (0.67)

## Data Availability

The original contributions presented in this study are included in the article and Supplementary Materials. Further inquiries can be directed to the corresponding author.

## Supplementary Materials

The supporting information can be downloaded at: https://www.mdpi.com/article/10.3390/jimaging12090462/s1.
