# Supplementary material for: Large Language Models Meet Gynecologic Ultrasound: Advancing the Characterization of ADNEXal Masses
Source: J Imaging. 2026 Sep 21;12(9):462. doi: 10.3390/jimaging12090462 (PMC13608372; doi:10.3390/jimaging12090462)
Supplement: Supplementary file 1 [file jimaging-12-00462-s001.zip › jimaging-4490658-supplementary-update.pdf]

Supplementary Material

Large Language Models Meet Gynecologic Ultrasound: Advancing the Characterization of ADNEXal Masses

Giulia Soccio [1], Stefania Di Napoli [1,*], Paolo Trerotoli [1], Vera Loizzi [2,3], Laura Grazia Zompì [1], Giuseppe Colonna [2], Daniele La Forgia [4], Gennaro Cormio [2,5] and Francesca Arezzo [2]

[1] Department of Interdisciplinary Medicine (DIM), University of Bari "Aldo Moro", 70124 Bari, Italy; giulia.soccio@uniba.it (G.S.); paolo.trerotoli@uniba.it (P.T.); laurazompi@uniba.it (L.G.Z.)
[2] Gynecologic Oncology Unit, IRCCS Istituto Tumori "Giovanni Paolo II", 70124 Bari, Italy; vera.loizzi@uniba.it (V.L.); g.colonna@oncologico.bari.it (Giuseppe Colonna); gennaro.cormio@uniba.it (Gennaro Cormio); f.arezzo@oncologico.bari.it (F.A.)
[3] Translational Biomedicine and Neuroscience Department (DiBraIn), University of Bari "Aldo Moro", Polyclinic of Bari, 70124 Bari, Italy
[4] SSD Radiologia Senologia, IRCCS Istituto Tumori "Giovanni Paolo II", 70124 Bari, Italy; d.laforgia@oncologico.bari.it
[5] Unit of Obstetrics and Gynecology, Department of Interdisciplinary Medicine (DIM), University of Bari "Aldo Moro", Polyclinic of Bari, 70124 Bari, Italy
* Correspondence: stefania.dinapoli@uniba.it; Tel.: +39-3893109116

1. Primary Analysis Prompt Schemes (Benign vs. Malignant)

All clinical questions were framed in English following standard IOTA terminology. Parameters were systematically presented in a fixed sequence: **morphology** → **lesion diameter** → **color score** → **additional clinical/ultrasound feature**.

Model A — Main Analysis (N = Entire Sample)

Predefined US Parameters

Parameters Included: Morphology, maximum diameter (mm), color score (1–4).

Question Scheme (Verbatim Example):

```
"Is a [morphology] ovarian cyst of [diameter] mm with a color score [color score] more likely benign or malignant?"
```

Model B — Main Analysis (N = Entire Sample)

Predefined US Parameters + Laboratory Data

Parameters Included: Morphology, maximum diameter (mm), color score (1–4), pre-surgical serum CA125 level (U/mL).

Question Scheme (Verbatim Example):

```
"Is a [morphology] ovarian cyst of [diameter] mm with a color score [color score] and a pre-surgical CA125 level of [CA125 value] U/mL more likely benign or malignant?"
```

Model C — Sub-Analysis (N = 135)

Solid & Multilocular Solid Morphologies + Clinical Feature

Parameters Included: Morphology (solid/multilocular solid), maximum diameter (mm), color score (1–4), presence/absence of ascites.

Question Scheme (Verbatim Example):

```
"Is a [solid / multilocular solid] ovarian cyst of [diameter] mm with a color score [color score] and [presence / absence] of ascites more likely benign or malignant?"
```

Model D — Sub-Analysis (N = 253)

Solid & Unilocular Solid Morphologies + US Feature

Parameters Included: Morphology (solid/unilocular solid), maximum diameter (mm), color score (1–4), presence/absence of acoustic shadows.

Question Scheme (Verbatim Example):

> "Is a [solid / unilocular solid] ovarian cyst of [diameter] mm with a color score [color score] and [presence / absence] of acoustic shadows more likely benign or malignant?"

Model E — Sub-Analysis (N = 134)

Unilocular Solid & Multilocular Solid Morphologies + US Feature

Parameters Included: Morphology (unilocular solid/multilocular solid), maximum diameter (mm), color score (1–4), presence/absence of vascularized papilla/e.

Question Scheme (Verbatim Example):

> "Is a unilocular solid ovarian cyst of 14 mm with a color score 2 and a vascularized papilla more likely benign or malignant?"

2. Secondary Analysis Prompt Scheme (Presumed Histological Diagnosis)

To evaluate the model's accuracy in predicting specific histopathology, standardized questions were constructed using comprehensive IOTA feature sets. Cystic content details were provided for unilocular/unilocular solid lesions; cystic content and locule count were added for multilocular/multilocular solid lesions.

Histological Diagnosis Prediction Model

Full Parameter Set

Parameters Included: Morphology, maximum diameter (mm), color score, presence/absence of acoustic shadows, presence/absence of ascites, serum CA125 level, cystic content (for unilocular/unilocular solid/multilocular/multilocular solid), and number of loci (for multilocular/multilocular solid).

Question Scheme (Verbatim Example):

> "Which is the histological presumed diagnosis for a unilocular solid ovarian cyst with the following features: diameter 199 mm, color score 2, anechoic content, CA125 of 31, no ascites, no acoustic shadows?"
